# Pilot Study of a New Mandibular Advancement Device

**DOI:** 10.3390/dj10060099

**Published:** 2022-06-06

**Authors:** Marzia Segù, Giovanna Campagnoli, Marco Di Blasio, Antonio Santagostini, Matteo Pollis, Luca Levrini

**Affiliations:** 1 Department of Medicine and Surgery, University of Parma, 43126 Parma, Italy; marzia.segu@unipr.it (M.S.); marco.diblasio@studenti.unipr.it (M.D.B.); 2 Department of Clinical-Surgical, Diagnostic and Pediatric Sciences, Section of Dentistry of Pavia, University of Pavia, 27100 Pavia, Italy; giovinacampa@gmail.com; 3 School of Dentistry, University of Siena, 53100 Siena, Italy; matteo.pollis@gmail.com; 4 Department of Human Sciences, Innovation and Territory, Postgraduate School of Orthodontics, School of Dental Hygiene, University of Insubria, 22100 Como, Italy; luca.levrini@uninsubria.it

**Keywords:** obstructive sleep apnea, mandibular advancement devices, polysomnography

## Abstract

This study was conducted to determine the efficacy of a customized mandibular advancement device (MAD) in the treatment of obstructive sleep apnea (OSA). Eight patients (M = 3; F = 5; mean age = 56.3 ± 9.4) with a diagnosis of OSA confirmed by polysomnography (PSG) were recruited on the basis of the following inclusion criteria: apnea-hypopnea index (AHI) > 5, age between 18 and 75 years, body mass index (BMI) < 25, and PSG data available at baseline (T0). All were treated with the new NOA^®^ MAD by OrthoApnea (NOA^®^) for at least 3 months; PSG with NOA in situ was performed after 3 months of treatment (T1). The following parameters were calculated at T0 and T1: AHI, supine AHI, oxygen desaturation index (ODI), percentage of recording time spent with oxygen saturation <90% (SpO2 < 90%), and mean oxygen desaturation (MeanSpO2%). Data were submitted for statistical analysis. The baseline values were AHI = 21.33 ± 14.79, supine AHI = 35.64 ± 12.80, ODI = 17.51 ± 13.5, SpO2 < 90% = 7.82 ± 17.08, and MeanSpO2% = 93.45 ± 1.86. Four patients had mild OSA (5 > AHI < 15), one moderate OSA (15 > AHI < 30), and three severe OSA (AHI > 30). After treatment with NOA^®^, statistically significant improvements in AHI (8.6 ± 4.21) and supine AHI (11.21 ± 7.26) were recorded. OrthoApnea NOA^®^ could be an effective alternative in the treatment of OSA: the device improved the PSG parameters assessed.

## 1. Introduction

Obstructive sleep apnea (OSA) is a breathing disorder characterized by repeated collapse, total or partial, of the upper airway during sleep [1]. A recent systematic review indicated that OSA may affect nearly 1 billion adults aged 30–69 years, and the number with moderate to severe OSA was estimated to be almost 425 million [1]. However, most cases of OSA remain undiagnosed and untreated [1]. 

Treatments for OSA include the use of continuous positive airway pressure (CPAP) devices that, by blowing air into the upper airway, reverse the collapse and end the apnea event [2]. Although CPAP remains the gold standard treatment in moderate-to-severe OSA, the use of a mandibular advancement device (MAD) is also considered effective, especially in mild-to-moderate OSA and in cases of low adherence to CPAP therapy [2]. A MAD increases the upper airway space and reduces the risk of obstruction by pulling the jaw and tongue forward. The method has been shown to reduce by 50% the number of PSG-detected apnea-hypopnea events per hour (i.e., the apnea-hypopnea index, AHI) and achieve higher patient compliance compared with CPAP [3]. 

A MAD can be prefabricated or custom-made. Customized devices are titratable and have been reported to show higher comfort and compliance [3,4,5,6]. They have also been shown to be more effective and stable [3,7,8]. Vanderveken et al., comparing the efficacy of a customized versus a prefabricated device, found greater compliance and effectiveness with the custom-made appliance, while the thermoplastic MAD did not obtain a therapeutic effect due to lack of retention and lower comfort for the patients [4].

In the present study, a new MAD is evaluated. MAD is a custom-made, two-piece device designed using CAD-CAM technology to be smaller and less cumbersome than existing devices. It is designed for the individual patient on the basis of a detailed mandibular kinematic evaluation. It allows titration of the protrusive movement and can be reinforced for patients with bruxism. Since the relationship between the degree of mandibular protrusion and mouth opening influences the efficacy of this treatment, MAD is designed to ensure effective protrusion associated with reduced muscular discomfort. 

This study aims to evaluate the effectiveness of new MAD in the treatment of OSA.

The null hypothesis is that the new appliance is not associated with a significant improvement of the selected PSG parameters.

## 2. Materials and Methods

### 2.1. Patient Selection

The study was approved by the Unit Internal Review Board (17-1023).

All the patients involved in the study were selected by a dentist with expertise in sleep medicine on the basis of medical, psychological, and dental criteria. Individuals aged between 18 and 75 years with a body mass index (BMI) < 25, an AHI > 5, and a PSG-confirmed diagnosis of mild to severe OSA were eligible; all had previously refused CPAP treatment [9].

All prospective participants underwent a complete history and physical examination. Those with an unsuitable stomatognathic situation (fewer than 8 teeth per arch, temporomandibular disorder, periodontitis), central sleep apnea, or cardiovascular diseases were excluded. Pregnancy (from the third month of pregnancy to three months after delivery) was a further exclusion criterion.

In order to evaluate patient satisfaction, each step of the protocol was followed by the administration of a detailed questionnaire collecting information about symptoms, perception of treatment efficacy, side effects (rated in terms of frequency and severity), and adherence to the treatment. The questionnaire also included the Epworth Sleepiness Scale and the Berlin Questionnaire to assess snoring, daytime sleepiness, fatigue, hypertension, and BMI. 

Baseline PSG (T0) was compared with PSG after 3 months with the appliance in situ (T1), focusing on the following PSG respiratory parameters: AHI (mean number of apnea and hypopnea events per hour of sleep), supine AHI (mean number of apnea and hypopnea events per hour of sleep in the supine position), oxygen desaturation index (ODI), mean oxygen saturation (MeanSpO2), and hypoxemia index, i.e., the percentage of recording time spent with oxygen saturation <90% (SpO2 < 90%).

Patients read and signed an informed consent document prior to being enrolled in this study.

**Sample:** Eight patients with symptomatic OSA met the study criteria and were enrolled. They were prevalently females (5 females, 3 males) and generally middle aged (mean age: 56.25 ± 9.75 years). OSA was mild (5 ≥ AHI < 15), moderate (15 ≥ AHI < 30), and severe (AHI ≥ 30) in 4, 1, and 3 patients, respectively. At T0, the patients’ mean respiratory variables were AHI: 21.33 ± 14.79, Supine AHI: 35.64 ± 12.80, ODI: 17.51 ± 13.5, SpO2 < 90%: 7.82 ± 17.08, MeanSpO2: 93.45 ± 1.86. 

### 2.2. Device

**Figure 1:**
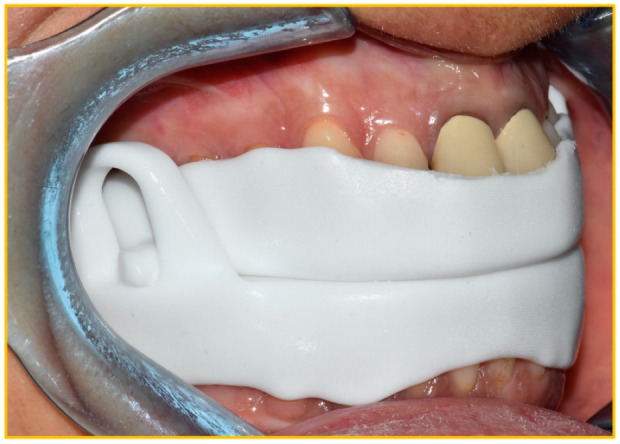
The device worn by the patient.

The oral appliance chosen for this study was NOA^®^ by OrthoApnea, a new titratable, custom-made, two-piece MAD with interconnected vertical extensions. The appliance comprises a maxillary bite and several mandibular bites that allow for sequential degrees of protrusion. The device is made of polyamide-12 through 3D printing with CAD-CAM technology, which allows reducing its size. NOA^®^ allows the patient a wide range of jaw movements (Figure 1).

### 2.3. Study Design

The first step of the protocol consisted of all the preliminary medical, dental, and neurological analyses, including evaluation of baseline PSG data and administration of questionnaires. TMJ clinical examination according to DC/TMD Axis I was performed [10]. Disease severity was defined by the AHI. 

In the second step, eligible patients were treated with NOA^®^ to test their tolerance of the device and, therefore, their likely response to this MAD therapy.

An initial habituation period was envisaged during which the patient kept the device in their mouth for short periods of time while awake. Thereafter, the device was put in at bedtime and worn throughout the night. Advancement (titration protocol) was progressively activated by the dentist until clinical resolution of subjective symptoms [11].

A follow-up PSG with NOA^®^ in situ was performed after 3 months of treatment [12,13].

**Statistical analysis**: Descriptive statistics (mean, standard deviation, median, minimum and maximum values) were calculated. The normality of the data was calculated using the Kolmogorov-Smirnov test. Subsequently, to analyze the results obtained with the NOA^®^, paired Student *t*-tests were performed to compare AHI, supine AHI, ODI, SpO2 < 90%, and MeanSpO2% between T0 and T1. For all tests, the level of significance was set at *p* < 0.05. 

## 3. Results

### Efficacy of the OrthoApnea NOA^®^ Device

Eight patients (3 males, 5 females; mean age: 56.25 ± 9.75 years) consented to use the device. Their respiratory index values at T0 were AHI: 21.33 ± 14.79, supine AHI: 35.64 ± 12.80, ODI: 17.51 ± 13.5, SpO2 < 90%: 7.82 ± 17.08 and MeanSpO2: 93.45 ± 1.86. Four had mild (5 ≥ AHI < 15), one moderate (15 ≥ AHI < 30), and three severe (AHI ≥ 30) OSA (Table 1). 

Two patients considered the device uncomfortable and decided not to continue with the therapy.

The remaining six patients were analyzed both at T0, before the treatment started, and after a minimum 3-month treatment with the device (T1) (Table 2).

**Figure 2:**
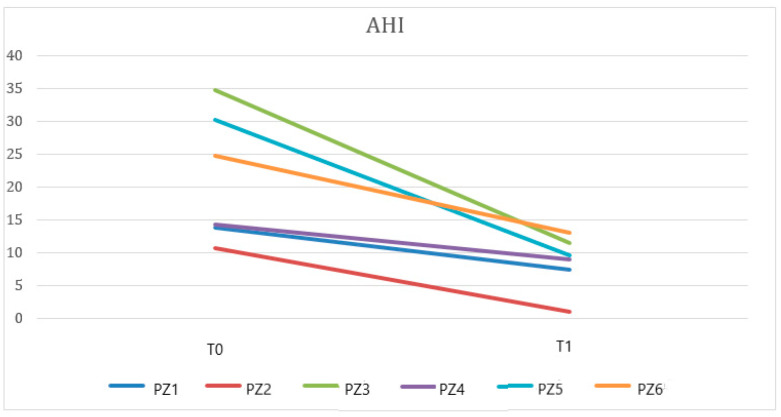
A paired data t-test showed a significant difference between AHI at T0 and T1 (*p* < 0.05) when evaluating data from patients (PZ) treated with the OrthoApnea NOA^®^ device.

**Figure 3:**
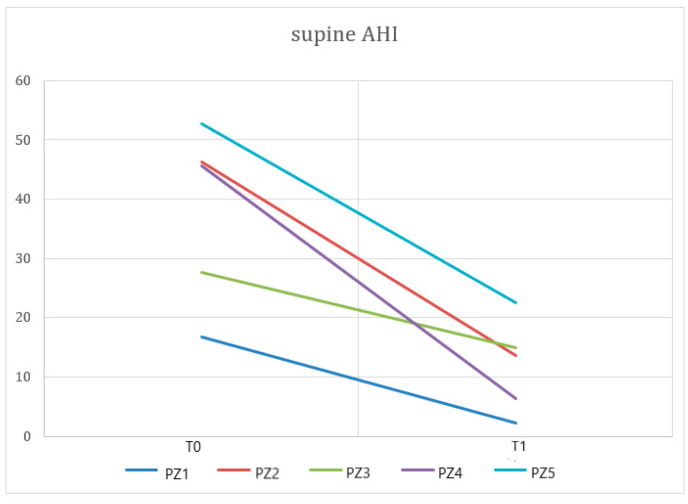
A paired data t-test showed a significant difference between supine AHI recorded at T0 and T1 (*p* < 0.05) in patients (PZ) using the OrthoApnea NOA^®^ device.

Paired *t*-tests showed statistically significant differences in AHI at T0 vs. T1 (Figure 2) or in supine AHI at T0 vs. T1 (*p* < 0.05) (Figure 3). No statistically significant differences were found in ODI, SpO2, and SpO2 < 90%. Table 2 details the short-term effects of treatment with NOA^®^ on the PSG respiratory parameters analyzed.

Regardless of the severity of their OSA, all six patients met the AHI and supine AHI criteria defining treatment success. In supine AHI, they showed reductions ranging from −17% to −86% and in AHI from −37% to −91% (Table 3).

One patient was reported to have AHI and supine AHI values < 5, which were considered non-pathological.

## 4. Discussion

Recent years have seen a growing interest in OSA in different fields of medicine due to its considerable prevalence [14] and its important role as a risk factor for cardiovascular and metabolic disorders [15]. 

Although CPAP remains the gold standard treatment for OSA [16,17], MAD treatment has emerged as an increasingly valuable alternative on account of its higher compliance and remarkable effectiveness in mild-to-moderate cases. MAD treatment is widely reported to reduce the severity of several OSA parameters in the long term, such as the AHI, ODI, and minimum oxygen saturation [18,19,20]. Improvements in daytime sleepiness and subjective perception of snoring were reported in several studies [8,21,22,23,24,25]. Although CPAP therapy was found to be more effective in reducing daytime sleepiness in the long term [24], MAD treatment achieved better therapeutic compliance [26,27,28]; instead, the two methods proved comparable in terms of improved quality of life, cognitive performance, and physical function, also in the long term.

For these reasons, further research into MAD treatment appears warranted. 

The wide variability in the response to treatment needs a strict control in each phase of the therapy [29,30,31]. The literature proposes different methods to distinguish responders and not responders, among which DISE-SAM protocol [32] and trial MAD [13]. Adequate follow-up [20,33,34] is essential for evaluating the efficacy of the therapy as well as for monitoring side effects related to long-term MAD use. The present study aims to test a definitive MAD that introduces the concept of maximum customization. NOA^®^, by OrthoApnea, is a two-piece, custom-made device designed using CAD-CAM technology. The appliance offers the possibility to titrate the protrusive movement, which is crucial for maximizing the therapeutic effect; furthermore, NOA^®^ is designed for the individual patient on the basis of a detailed mandibular kinematic evaluation in order to achieve maximum comfort and compliance.

The results of our study showed statistically significant changes in AHI and supine AHI values at T1 compared with T0 (*p* < 0.05). Conversely, no statistically significant changes were found in ODI scores (*p* > 0.05), SpO2 < 90%, or MeanSpO2% (*p* > 0.05) (Table 2).

According to the literature, a MAD can be considered effective if it leads to an at least 50% reduction in the AHI [21,35]. The NOA^®^ by OrthoApnea met this criterion, leading to statistically significant improvements (reductions) of around 59% in both AHI and supine AHI. A review by Marklund et al. [21] found that MAD treatment reduced AHI values by at least 50%: the treatment was deemed successful (success being defined as an AHI < 5) in 17–75% of patients, while AHI values < 10 were reported in 30–94% of the patients in all the studies considered. De Britto Texeira and colleagues [36] reported that thanks to the use of a suitably modified twin block device, 47% of their OSA sample obtained a 50% reduction in AHI, while in 26%, the parameter returned to normal values. In a study conducted by Duràn-Cantolla [24], 47% of the sample obtained a significant reduction in AHI. Overall, the patients in this study quickly adapted to the NOA^®^; in one case, treatment adherence was undermined by discomfort associated with the device, while a further patient used the device incorrectly and therefore failed to follow the therapeutic protocol. These findings underline the importance of considering the influence of patient-dependent variables, above all compliance, which is decisive in achieving therapeutic success.

The literature agrees that the side effects initially associated with MAD use are mostly transient and can be resolved simply by modifying certain features of the device. In several studies, the side effects associated with the initial phase of MAD therapy did not lead to discontinuation of the treatment by the patient. In the sample examined by Milano and colleagues [25], for example, some patients experienced side effects in the first month of treatment, such as temporomandibular joint discomfort, difficulty chewing in the morning, and dental tenderness; however, none of these problems precluded continued use of the device.

The literature shows that personalized devices, such as NOA^®^ by OrthoApnea, are associated with greater patient-perceived comfort and better therapeutic outcomes than prefabricated devices, these better outcomes being a result of greater adherence to the treatment. According to an AADMS (American Academy of Dental Sleep Medicine) report, systematic reviews have shown that personalized devices compare better to prefabricated non-customizable ones. Partly because they provide more significant and better retention, they are able to keep the jaw in a more stable position; consequently, they are more effective and comfortable [37]. Guidelines on the clinical management of OSAS with oral devices, published by the AADMS [3], recommend the prescription of a personalized and titratable MAD rather than the prefabricated, non-titratable type. The literature evidence shows that the former type is more effective than the latter in improving AHI and other cardiorespiratory parameters. The 2014 TOMADO crossover study [5] compared the efficacy of three different types of monoblock MAD in a sample of 90 patients. Although the three types gave positive and comparable results, the non-personalized thermoplastic device was the least comfortable due to poorer retention; this led to lower therapeutic adherence compared with the semi-personalized and personalized devices.

In our study, 75% of the sample treated with NOA^®^ accepted the device and followed the treatment protocol without experiencing the problems commonly related to the use of MADs. This could be due to the refined, customized design of this device outlined above. However, to confirm this scientifically, it would be necessary to compare NOA^®^ with other custom-made MADs and enlist a larger sample of patients.

Mandibular advancement devices are capable of improving health and quality of life, including social life [6,26], and thanks to their therapeutic efficacy in treating severe OSAS, MAD therapy is suitable for all patients refusing CPAP. A meta-analysis conducted by Schwartz [26] in 2018 showed a difference in treatment compliance between the two therapies: with CPAP, it was 1.1 h per night lower than with MAD (*p* = 0.004). This explains why CPAP does not show significant quality-of-life improvements compared with MAD therapy, specifically significantly improved cognitive and functional results. This is despite CPAP being more effective in improving cardiorespiratory parameters. 

The present study does not have enough data to obtain statistically significant results regarding the follow-up of the patients beyond the 3-month (T1) assessments. However, numerous studies in the literature attest to the effectiveness of personalized MADs in improving OSAS symptoms over time [18,19,21,31]. The most significant studies in this regard include one conducted by Uniken Venema and colleagues in 2020 [20]. These authors set out to compare the long-term effectiveness of CPAP and MAD in patients with OSAS through evaluation of the effects of treatments over ten years. Polysomnography results showed a favorable outcome of both therapies at ten years: the mean AHI in the MAD group was 9.9 ± 10.3 events/h, versus 3.4 ± 5.4 events/h in the CPAP group. Both therapies led to a substantial improvement in self-reported neurobehavioral outcomes at ten years of follow-up. Attali and colleagues [19] also showed that MADs can effectively treat OSAS in the long term, maintaining good compliance and patient satisfaction. Their study, conducted in a sample of 279 patients with an average age of 58 years and followed up for at least 1000 days, showed that 63% of the sample (at 2.5 years) continued treatment with MAD with adequate efficacy, tolerability, and compliance over time; only in some patients was a recurrence of side effects observed, probably due to the natural course of the disease or to wear and tear of the MAD associated with a loss of therapeutic efficacy.

### Limitations and Future Perspectives

Our study was limited mainly by the short follow-up time, the small sample size and the lack of a control group (no treatment/other MAD). These limitations might be overcome by recruiting a larger sample and extending the therapeutic monitoring time, controlling for a series of time-dependent variables. Since OSAS is a chronic disease that can worsen over time, it is important to maintain a constant improvement as the treatment progresses; OSAS is also a risk factor for a series of systemic diseases, meaning that patients’ general health also needs to be monitored. In some cases, clinicians may consider reinforcement of behavioral therapy and sleep hygiene measures in order to address negative habits.

In addition, since there is no standardized titration protocol, it would be appropriate in future research to evaluate whether the level of mandibular advancement determined by the MAD corresponds to the individual therapeutic window. Dieltjens et al. [30] conducted an exhaustive review of the titration techniques in use, especially the “trial and error” method that is currently the most popular. This method involves selecting a particular mandibular protrusion setting and evaluating its side effects and associated benefits, thereafter reaching the individual therapeutic window by process of trial and error. With the OrthoApnea NOA^®^ system, too, the patient is required to follow a protrusive sequence established in advance, in this case, on the basis of individual variables. The device includes a series of lower splints that reproduce the established protrusive line, allowing the patient gradually to reach his/her own most effective level of mandibular advancement. To motivate the patient and enhance the therapeutic alliance, and therefore allow the therapeutic window to be reached, the personalized titration protocol should be carefully explained to the patient.

Finally, the device requires some maintenance since it can deteriorate over time, leading to a reduction in retention, comfort, and therapeutic efficacy. Again, to maintain patient compliance and the effectiveness of the treatment, periodic re-evaluation of the device is recommended so that any necessary modifications or replacements can be made.

## 5. Conclusions

Treatment with NOA^®^ could be an effective alternative in the treatment of OSAS, having been found to significantly improve some PSG respiratory parameters, specifically AHI and supine AHI, even in cases of severe OSAS (AHI > 15). The other parameters investigated (ODI, SpO2 < 90%, and MeanSpO2) also improved, albeit not significantly. In conclusion, although the statistical data are promising, to establish the potential of the device, further studies are needed.

## Figures and Tables

**Table 1:** Characteristics of the whole population at baseline (T0).

Number of patients	8
Males	3
Females	5
Age, mean ± SD	56.25 ± 9.75
OSAS severity, number of patients	
Mild (5 ≥ AHI < 15)	4
oderate (15 ≥ AHI < 30)	1
Severe (AHI ≥ 30)	3
AHI	21.33 ± 14.79
Supine AHI	35.64 ± 12.80
ODI	17.51 ± 13.5
SpO2 < 90%	7.82 ± 17.08
MeanSpO2%	93.45 ± 1.86

**Table 2:** Short-term effects of the appliance on respiratory variables in the six patients who continued with the therapy.

	T0	T1	*p*
AHI	21.33 ± 9.75	8.6 ± 4.21	0.008
Supine AHI	35.64 ± 12.80	11.21 ± 7.26	0.002
ODI	17.51 ± 13.5	8.81 ± 4.59	0.07
SpO2 < 90%	7.82 ± 17.08	0.66 ± 1.43	0.17
MeanSpO2	93.45 ± 1.86	93.66 ± 1.03	0.82

**Table 3:** Short-term effects of the appliance: changes in AHI and supine AHI.

Patients	Changes in AHI T0–T1	Changes in Supine AHI T0–T1
1	−46%	−75%
2	−91%	−86%
3	−67%	−17%
4	−37%	−46%
5	−68%	−86%
6	−47%	−57%

## Data Availability

All data are available upon request to the corresponding author.
